# Short-term effects of meteorological factors on pediatric hand, foot, and mouth disease in Guangdong, China: a multi-city time-series analysis

**DOI:** 10.1186/s12879-016-1846-y

**Published:** 2016-09-29

**Authors:** Cui Guo, Jun Yang, Yuming Guo, Qiao-Qun Ou, Shuang-Quan Shen, Chun-Quan Ou, Qi-Yong Liu

**Affiliations:** 1 State Key Laboratory of Organ Failure Research, Department of Biostatistics, Guangdong Provincial Key Laboratory of Tropical Disease Research, School of Public Health, Southern Medical University, Guangzhou, 510515 Guangdong China; 2 State Key Laboratory of Infectious Disease Prevention and Control, Collaborative Innovation Center for Diagnosis and Treatment of Infectious Diseases, National Institute for Communicable Disease Control and Prevention, Chinese Center for Disease Control and Prevention, Beijing, 102206 China; 3 Division of Epidemiology and Biostatistics, School of Public Health, The University of Queensland, Brisbane, QLD 4006 Australia; 4 Department of Pediatrics, Guangzhou First People’s Hospital, Guangzhou Medical University, Guangzhou, Guangdong 510180 China

**Keywords:** Hand, foot, and mouth disease, Meteorological factors, Mixed generalized additive model, China

## Abstract

**Background:**

Literature shows inconsistency in meteorological effects on Hand, foot, and mouth disease (HFMD) in different cities. This multi-city study aims to investigate the meteorological effects on pediatric HFMD occurrences and the potential effect modification by geographic factors.

**Methods:**

Based on daily time-series data in eight major cities in Guangdong, China during 2009–2013, mixed generalized additive models were employed to estimate city-specific meteorological effects on pediatric HFMD. Then, a random-effect multivariate meta-analysis was conducted to obtain the pooled risks and to explore heterogeneity explained by city-level factors.

**Results:**

There were a total of 400,408 pediatric HFMD cases (children aged 0–14 years old) with an annual incidence rate of 16.6 cases per 1,000 children, clustered in males and children under 3 years old. Daily average temperature was positively associated with pediatric HFMD cases with the highest pooled relative risk (RR) of 1.52 (95 % CI: 1.30–1.77) at the 95th percentile of temperature (30.5 °C) as compared to the median temperature (23.5 °C). Significant non-linear positive effects of high relative humidity were also observed with a 13 % increase (RR = 1.13, 95 % CI: 1.00–1.28) in the risk of HFMD at the 99th percentile of relative humidity (86.9 %) as compared to the median value (78 %). The effect estimates showed geographic variations among the cities which was significantly associated with city’s latitude and longitude with an explained heterogeneity of 32 %.

**Conclusions:**

Daily average temperature and relative humidity had non-linear and delayed effects on pediatric HFMD and the effects varied across different cities. These findings provide important evidence for comprehensive understanding of the climatic effects on pediatric HFMD and for the authority to take targeted interventions and measures to control the occurrence and transmission of HFMD.

**Electronic supplementary material:**

The online version of this article (doi:10.1186/s12879-016-1846-y) contains supplementary material, which is available to authorized users.

## Background

Hand, foot, and mouth disease (HFMD) is a common viral illness that usually affects infants and children under five, characterized with typical symptoms of fever, skin eruptions on hands and feet, and vesicles in oropharynx [1]. Because of non-effective therapy or vaccine, there is wide transition and periodical outbreak. Most cases are mild and self-limited, however, rare but severe complications or even deaths may occur. HFMD burdens the entire society and constitutes a worldwide public health threat. Particularly in recent years, the morbidity of HFMD keeps growing. In mainland China, HFMD is monitored as a class “C” notifiable disease with 2,712,925 reported cases and 384 deaths in 2014 [2]. Guangdong, the biggest province in South China, has suffered frequently from HFMD epidemics, because of the large population density and mobility and the typical tropical or sub-tropical climate.

Previous literature has shown that HFMD morbidity has a 1-year periodicity, with a major peak between spring and early summer and a smaller peak during autumn [3–5]. Some single-city studies have documented that meteorological factors play an important role in the seasonal pattern of HFMD, but there are inconsistencies in the identified factors, the exposure-response relationships, the lag patterns and the time scales. For example, Chang et al [6] found HFMD cases increased with daily mean temperature in Taiwan, while such effects were not found in island-type territory in East Asia [7]; Wu et al [8] pointed out a non-linear relationship between humidity and HFMD cases in Rizhao, China, but a positive linear association was identified in Taiwan [6]; The maximum lag of delayed meteorological effects was identified from several days to 3 months [8, 9].

Previous assessments of meteorological effects on HFMD occurrences were mainly based on an Autoregressive Integrated Moving Average (ARIMA) model and Seasonal Autoregressive Integrated Moving Average (SARIMA) model, which clarify the effects of meteorological factors at temporal scale with the hypothesis of linear association [9, 10]. It is required to further explicate potential lag patterns and nonlinear features of meteorological effects. Recently, a few studies have applied generalized additive model (GAM) or distributed lag non-linear models (DLNM) to quantify the temporal effects of meteorological factors on HFMD [10, 11]. In addition to temporal variations, there were substantial geographic differences in HFMD occurrences, even in the same climate [12], however, the influence of geographic factors on the climate-HFMD association remains unknown.

This study aims to investigate the meteorological effects on pediatric HFMD occurrences, and to examine potential effect modification by geographic factors in eight major cities in Guangdong, China.

## Methods

### Data sources

**Fig. 1:**
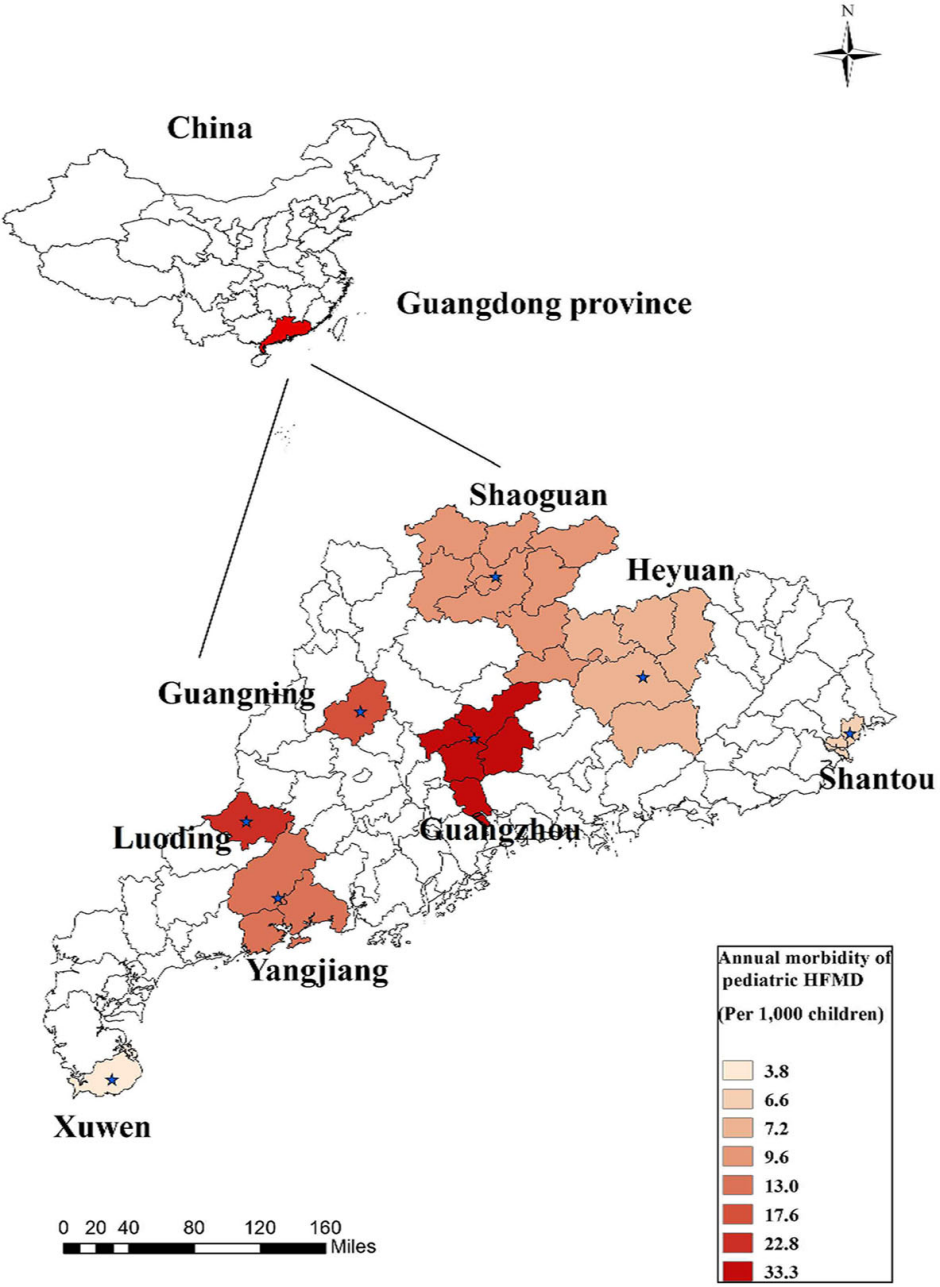
Map of Guangdong and annual morbidity of pediatric HFMD (per 1,000 children) in eight cities. The stars represent the locations of the cities

In mainland China, the web-based infection diseases monitor information system has been established in 2004, and HFMD has been monitored and reported as a class C notifiable disease since 2008. This system was managed and maintained by the Chinese Centre for Disease Control and Prevention (China CDC). China CDC provided all pediatric HFMD cases (patients aged 0–14 years old) in eight major cities of Guangdong Province from 1 January 2009 to 31 December, 2013. The eight cities included Guangzhou, Shaoguan, Shantou, Heyuan, Yangjiang, Guangning, Luoding, and Xuwen (Fig. 1).

Daily meteorological data during the study period for each city were obtained from the China Meteorological Data Sharing Service System, including daily average temperature, precipitation, atmospheric pressure, relative humidity, wind speed and sunshine hours. Demographical and socio-economic data in each city, including population density (persons per square km), the ratio of male to female population (%), the ratio of children under 5 years old (%), Gross Domestic Product (GDP) per capita (RMB), living space per capita (square meter), and the ratio of population flow (%) were collected from the Sixth National Population Census of China in 2010.

### Statistical analyses

A two-stage analysis was performed to quantify the meteorological effects on HFMD occurrences. In the first stage, Spearman rank correlation analysis was conducted to check the collinearity between meteorological factors. There was a strong negative correlation between atmospheric pressure and average temperature with a Spearman correlation coefficient ranging from -0.885 to -0.821 in eight cities, and precipitation was highly correlated with relative humidity (Additional file 1). Considering no evidence of the effects of atmospheric pressure and precipitation on HFMD, and avoiding a collinearity problem, we did not include atmospheric pressure and precipitation in the final model.

A mixed generalized additive model (MGAM) was used for each of eight cities to derive parameter estimates of the exposure–response associations between meteorological factors and HFDM. In the second stage, these city-specific estimates were pooled as outcomes using a random-effect multivariate meta-analytical model. We examined the potential heterogeneity between cities and identified city-level factors associated with the heterogeneity.

#### First-stage model

As an extension of GAM, MGAM incorporates additive parametric functions of covariates and autoregressive terms into models [13]. MGAM has very good performance to control for the autocorrelation of residuals and lead to a robust estimate of the estimates and standard errors. We firstly examined the effects of four meteorological measures and did not find statistically significant effects of average wind speed and sunshine hours on HFMD (Additional file 2). To avoid the redundancy or the inappropriate parameters estimates, these two factors were excluded from the final model. That is, we only considered the effects of mean temperature and relative humidity in the final model. The first-stage model can be specified as follows:

1 $$ \ln \left(\mathrm{E}\left({y}_{it}\right)\right) = {l}_{it} + {\tau}_{it} $$

fixed effects:

2 $$ \begin{array}{l}\begin{array}{l}{l}_{it}=f\left(tem{p}_{it},4\right)+f\left( hum{i}_{it},4\right)\hfill \\ {}\kern3em  + ns\left(tim{e}_i,5*6\right)+I\left( Holida{y}_{it}\right)+I\left( DO{W}_{it}\right)\hfill \end{array}\\ {}\ \end{array} $$

autoregressive terms:

3 $$ {\tau}_{it}={\displaystyle \sum_{\mathrm{k}=1}^3}{\beta}_{ik}\left( \ln \left({\mathrm{y}}_{i,t-k}^{*}\right)-{l}_{i,t-k}\right) $$

where y^*^_i,t-k_ = max (*y*_*i,t-k*_, 0.5) and *β*_*ik*_ is the coefficient of autoregressive random effect. *y*_*it*_ is the daily number of HFMD cases in city *i* (1,2, …, 8) on calendar day *t* (1, 2, …,1826), supposed to follow the quasi-Poisson distribution allowing for over-dispersion. Function *f* S_j_ represents the two-dimensional space of cross-basis functions of natural cubic splines with unified lag to determine their distributed lag non-linear effects [14, 15]. *Temp*_*it*_ and *humi*_*it*_ indicate daily average temperature and relative humidity with 4° of freedom (*df*). Consist with previous study, we specified a maximum lag of 14 days [16] to adequately examine the lagged effects. A natural cubic spline of *time* with 6 *df* per year was applied to control for he seasonality and long-term trend of HFMD occurrences. *Holiday* and *DOW* are the categorical variables indicating the public holiday and day of the week, respectively. The relative risk (RR) of HFMD occurrences was calculated as compared to the median values of meteorological factors.

To further examine whether the meteorological effects on HFMD differed in two peak seasons (i.e. spring and autumn), we conducted stratified analyses for spring (March-May) and autumn (September-November). Sensitivity analyses were performed by varying *df* (3–5) for lags, *df* (3–5) for meteorological factors, *df* (4–7) for time as well as the maximum lag (14–16).

#### Second-stage model

The second-stage model can be specified as follows [17]:

4 $$ {\theta}_i^{\hbox{'}}\sim {\mathrm{N}}_k\left(\theta, {S}_i+\varPsi \right) $$

where *θ* is the coefficients of the function *f. θ*_*i*_ is the valid parameter in city *i. θ*^*’*^_*i*_ is the estimate of *θ*_*i*_ as the outcome parameters acquired in the first stage and follows the multivariate normal distribution N_k_(*θ*_*i*_, *S*_*i*_) with *k* dimension. *Ψ* represents the between-city variance-covariance matrix. The restricted maximum likelihood (REML) was utilized to estimate the parameters in the random-effect multivariate meta-analytical model. The heterogeneity was identified by the Cochran Q test and quantified by I^2^. Best linear unbiased prediction (BLUP) was applied to project the exposure–response associations [18]. Wald test was used to identify the statistical significance of the associations between the city-level variables and the outcome parameters. All data analyses were performed in R 3.1.2.

## Results

During the 5-year period from 1 January, 2009 to 31 December, 2013, there were a total of 400,408 HFMD cases, with an annual morbidity of 16.6 cases per 1,000 children. The majority of cases (95.3 %) were diagnosed by clinical symptoms and signs. The most common causative agent are *coxsackievirus A16* (*CV-A16*) (4855 cases, 25.8 %) and *enterovirus71* (*EV71*) (4407 cases, 23.4 %). 397 cases (0.1 %) experienced severe situation among which a considerable proportion of patients (14.4 %) died. The annual morbidity among male was approximately 1.5 times as high as that among female. Children under 3 years old were at the highest risk of HFMD with an annual morbidity of 58.0 cases per 1,000 children. Most cases (80.11 %) were scattered children who did not attend school or kindergarten, and among them 93.01 % were under 3 years old. The summarized statistics of HFMD cases and meteorological factors in each city were shown in Additional file 3.

**Fig. 2:**
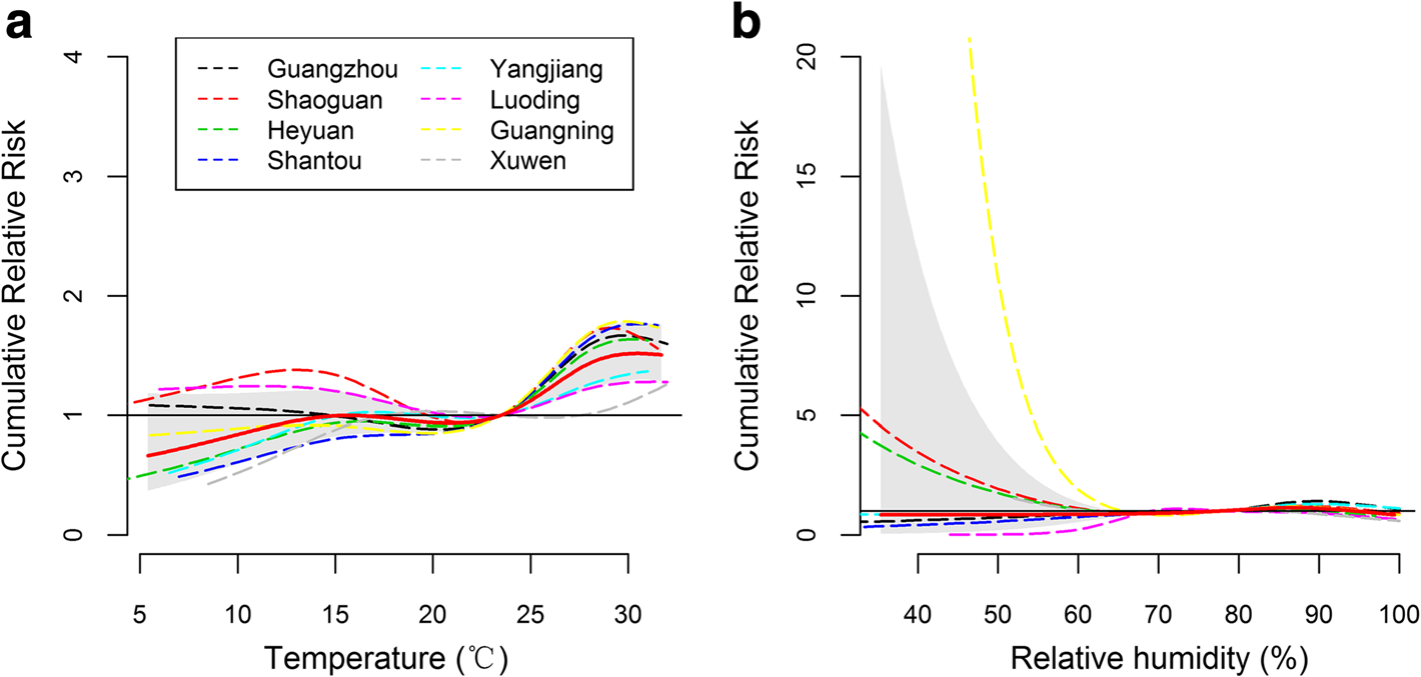
The cumulative effects of meteorological factors on HFMD over lag 0–14 days in Guangdong, 2009–2013. **a** and **b** show the cumulative relative risks of HFMD associated with temperature and relative humidity, respectively. The bold red line represents the pooled effects, and the dashed lines represent the city-specific estimates. Reference values were the medians, that is, 23.5 °C for temperature and 78.0 % for relative humidity

**Fig. 3:**
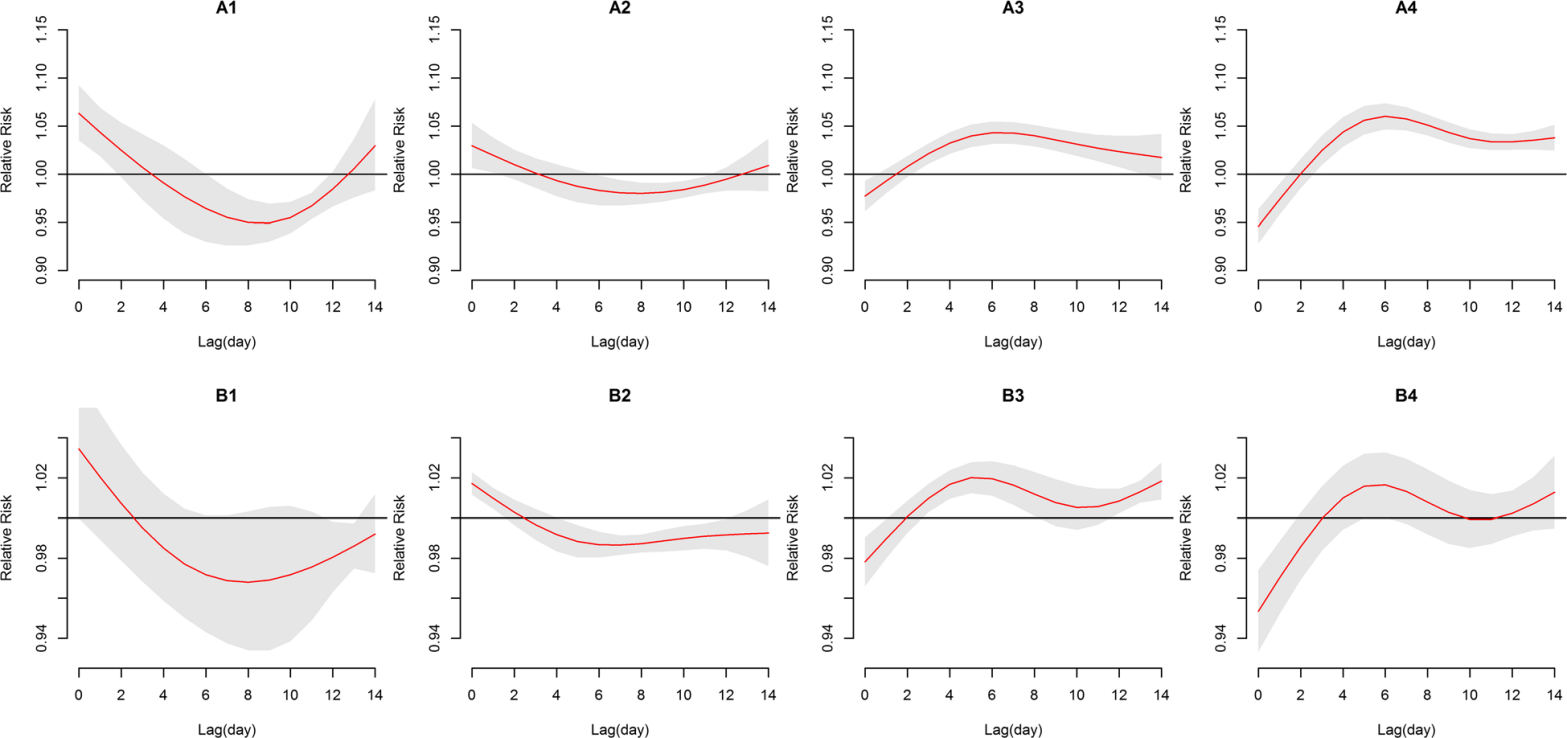
The lag patterns of pooled effects of meteorological factors on HFMD across lag 0–14 days. A1-A4 show the pooled relative risks (RRs) of HFMD associated with the 5th, 25th, 75th, and 95th percentiles of average temperature compared to the median temperature, respectively. B1-B4 show the corresponding effects of relative humidity

Figure 2 shows the exposure-response associations between meteorological factors and HFMD cases in each of eight cities. Although there were some differences in the magnitude of effect estimates (i.e. RR) among eight cities (Fig. 2a), in general, daily average temperature was positively associated with HFMD. The highest pooled cumulative RR was 1.52 (95 % confidence interval (CI): 1.30–1.77) over lag 0–14 days at the 95th percentile (30.5 °C) as compared to the median temperature (23.5 °C). The effects of high temperatures peaked at lag 6 days and nearly disappeared at lag 14 days (Fig. 3-A3 and 3-A4). There was 37 % increase (RR = 1.37, 95 % CI: 1.18–1.59) in HFMD cases over lag 0–14 days by comparing the 75th percentile of temperature to the median temperature, and significant cumulative effect estimates were also observed for lag 0–7 days. The risk of HFMD increased 19 % (RR = 1.19, 95 % CI: 1.07–1.31) and 51 % (RR = 1.51, 95 % CI: 1.29–1.77) over lag 0–7 days and 0–14 days, respectively, by comparing the 95th percentile to the median temperature (Table 1).

**Table 1:** The pooled effects of meteorological factors on HFMD occurrences in eight cities

	Relative Risk (95 % confidence interval)				
	Lag 0	Lag 7	Lag 14	Lag 0–7	Lag 0–14
Temperature (°C, reference at 23.5 °C)^a^					
9.8 (P5)	1.06 (1.04, 1.09)^b^	0.96 (0.93, 0.98)^b^	1.03 (0.98, 1.08)	1.02 (0.82, 1.28)	0.83 (0.59, 1.18)
17.4 (P25)	1.03 (1.01, 1.05)^b^	0.98 (0.97, 0.99)^b^	1.01 (0.98, 1.04)	0.98 (0.90, 1.07)	0.98 (0.85, 1.13)
27.3 (P75)	0.98 (0.96, 0.99)^b^	1.04 (1.03, 1.05)^b^	1.02 (0.99, 1.04)	1.16 (1.08, 1.26)^b^	1.37 (1.18, 1.59)^b^
29.7 (P95)	0.95 (0.93, 0.96)^b^	1.06 (1.05, 1.07)^b^	1.04 (1.02, 1.05)^b^	1.19 (1.07, 1.31)^b^	1.51 (1.29, 1.77)^b^
Relative humidity (%, reference at 78 %)^a^					
57.0 (P5)	1.03 (1.00, 1.07)	0.97 (0.94, 1.00)	0.99 (0.97, 1.01)	0.96 (0.69, 1.35)	0.85 (0.39, 1.83)
70.3 (P25)	1.02 (1.01, 1.02)^b^	0.99 (0.98, 0.99)^b^	0.99 (0.98, 1.01)	0.96 (0.90, 1.03)	0.90 (0.82, 0.99)^b^
85.0 (P75)	0.98 (0.97, 0.99)^b^	1.02 (1.01, 1.03)^b^	1.02 (1.01, 1.03)^b^	1.03 (1.00, 1.06)^b^	1.12 (1.02, 1.24)^b^
94.0 (P95)	0.95 (0.93, 0.97)^b^	1.01 (1.00, 1.03)	1.01 (0.99, 1.03)	1.02 (0.95, 1.09)	1.01 (0.85, 1.19)

^a^The reference value is the median for each meteorological measures

^b^Confidence intervals that do not overlap the null value of RR = 1

The cumulative RR of HFMD increased with the relative humidity and peaked at a relative humidity of the 99th percentile of humidity (86.9 %) with a RR of 1.13 (95 % CI: 1.00–1.28) compared to the median humidity (Fig. 2b). The lag-response curve shows that low humidity was associated with a decrease in HFMD occurrences (Fig. 3-B1 and 3-B2). High relative humidity was associated with an increase in HFMD and the effects grew during the lag period of 0–6 days (Fig. 3-B3 and 3-B4). An increase of 3 % (RR = 1.03, 95 % CI: 1.00–1.06) and 12 % (RR = 1.12, 95 % CI:1.02–1.24) in HFMD cases was associated with the 75th percentile compared to the median humidity over lag 0–7 days and lag 0–14 days, respectively (Table 1).

The HFMD incidences showed slightly differences with day of the week. An increase of 3.2 % (RR = 1.032, 95 % CI: 1.00, 1.065) was found in HFMD cases on Monday as compared to Sunday. The RRs (95 % CI) were 0.996 (0.96–1.025), 0.977 (0.957–0.998), 0.961 (0.923–0.999), 0.975 (0.926–1.027), and 0.96 (0.936–0.984) on Tuesday-Saturday, respectively, as compared to Sunday. HFMD incidence tended to be higher on holidays (RR = 1.02, 95 % CI: 0.960–1.084) than non-holidays.

A significant heterogeneity (Q = 75.93, *p* < 0.001) was identified between the eight cities in the intercept-only random-effect multivariate model, which contributed a moderate part (63.12 %) to the overall variations (Table 2). Multivariate meta-regression analyses revealed that the heterogeneity was associated with city’s latitude and longitude, while no significant association was found with other factors (*P* > 0.05) (Table 2). The inclusion of these two factors in the model showed a substantial decrease in heterogeneity (31.38 %), compared to the intercept-only model.

**Table 2:** Heterogeneity of meteorological effects and its association with city-level factors

Study-level variables	Cochran Q test		I^2^	Information criteria		Wald test	
	Q	P	%	AIC	BIC	Stat	P
Intercept-only	75.93	<0.001	63.12	66.67	85.32	-	-
Geographically factors							
Latitude	45.19	<0.001	46.89	67.08	88.29	26.58	<0.001
Longitude	57.60	<0.001	58.34	78.88	100.09	17.60	<0.001
Height	70.45	<0.001	65.93	106.96	128.16	2.09	0.72
Demographic factors							
Population density	66.15	<0.001	63.72	127.10	148.30	4.73	0.32
The ratio of male to female	73.47	<0.001	67.33	91.38	112.59	2.37	0.67
Ratio of children under five	68.90	<0.001	65.16	85.37	106.58	1.50	0.83
Socio-economic factors							
GDP per capita	68.19	<0.001	64.80	160.55	181.76	4.61	0.33
Living space per capita	69.82	<0.001	65.63	91.71	112.92	3.33	0.50
Ratio of population flow	66.72	<0.001	64.03	100.33	121.53	4.63	0.33
Significant factors							
Latitude and longitude	29.30	0.08	31.74	82.22	104.13	40.45	<0.001

The stratified analyses revealed that HFMD risk increased with temperature and relative humidity both in spring and autumn, and there were generally not substantial differences in the effect estimates (Additional file 4). Sensitivity analyses on *df* for meteorological factors, lag, and time, and maximum lag demonstrated that the final model was appropriate and stable without substantial variations in Likelihood Akaike information criteria for quasi-Poison (Q-AIC) value and the effect estimates.

## Discussion

We observed the annual morbidity of pediatric HFMD was 16.6 cases per 1,000 children in eight cities in Guangdong, China. The HFMD morbidity in the whole population in Guangdong (2.2 cases per 1,000 persons) was approximately 1.6 times higher than the national average in China (1.4 cases per 1,000 persons) in 2010 and 2.8 times than that reported in Japan in 2010 (0.8 cases per 1,000 persons) [3, 19, 20]. The morbidity of HFMD in males was approximately 1.5 times as high as that among females, consistent with the reports in Korea and Japan [21, 22]. A possible explanation could be gender difference in susceptibility at host genetic level [23]. Also, children under three years old were more susceptible to HFMD, which was observed in other regions [24, 25]. Their poor immune systems that are lack of neutralizing antibodies can also contribute to high susceptibility to HFMD infection [26]. By contrast, a study in Taiwan found that infant had the least HFMD morbidity probably because of the preexisting neutralizing antibody to *EV71*, acquired through transplacental transfer [27].

In this study, we observed non-linear and delayed effects of daily average temperature, and relative humidity on HFMD cases, and such effects varied geographically. There is no doubt that temperature, especially the average temperature, is the generally acknowledged meteorological factor affecting HFMD occurrences [10, 11, 19, 28]. Several researches considered maximum or minimum temperature as an indicator [10, 19, 29], but average temperature could be the most familiar and powerful predictor to the public. The positive and significant non-liner effects of average temperature on HFMD cases were found in this study, supported by preceding findings [10, 19, 22, 29]. The effects peaked at 30.5 °C (the 98th percentile of temperature), which was similar to the finding in Japan (about 29 °C, the 95th percentile of temperature) [22]. This is reasonable according to an experimental finding in cynomolgus monkeys that the infectivity and activity of *EV71* could be inhibited when the temperature is higher than 25 °C due to the potential properties of circulating strains [30, 31]. Besides, a vitro experiment noted that *enterovirus* replication was restricted approximately 90 % at 39 °C, as compared to the replication at 37 °C [32]. Additionally, the serological antibody in human body may be adjusted by temperature. An experimental study in mice has shown that the antibody to *EV71* increases with temperature by accelerating the DNA replication [33], suggesting that high ambient temperature may limit HFMD occurrences. However, the mechanism still needs to be explored in future researches.

Relative humidity was another significant factor positively affecting the HFMD occurrences and the associated risk did not decrease until it was 86.9 % (the 99th percentile of humidity). The potential reason for the effects of relative humidity is possibly due to its profound effects on immunity-oriented problems. The metabolism rate of children decrease when the relatively humidity is relatively high, facilitating the HFMD infection. Besides, previous experiment found that under high relative humidity, *enterovirus 70* can be recovered from non-porous surfaces even after 24 h and high humid condition was important for the transmission of *enterovirus 70* [34]. We observed high relative humidity effect delayed 2 days and peaked at about 6 days later, which was supported by the possible period of HFMD incubation (2–7 days). A longer lag up to 3 weeks was recognized in Japan despite its insignificant effect [22]. Limited literature has demonstrated the potential explanation of this discrepancy in lag patterns. The longer lag days seemed to be irrational considering the incubation period.

In this study, we found that Sunday and Monday had relatively higher HFMD incidence than the rest days of the week. The HFMD incidence tended to be higher on holidays than on non-holidays. Ma et al [13] pointed out a similar trend in bacillary dysentery incidence. This can be explained by patients’ behaviors. On public holidays, children have more outdoor activities and cluster in public places, which could increase the infection rate of HFMD. Moreover, infected children are more likely to be taken to see doctors on weekends rather than on weekdays.

There are substantial geographic variations in HFMD. Having an understanding of the geographical heterogeneities of meteorological effects between different areas may provide an appropriate way to estimate HFMD risks and powerful evidence for local authorities to establish precise warming system of HFMD occurrences. A study in East Asia observed variations in peak timing of HFMD occurrences among cities at different latitudes [35], which was caused mainly by meteorological factors. Hu et al [36] found that the morbidity of HFMD was significantly associated with population density and meteorological factors based on a Geographically Weighted Regression Model. However, to our knowledge, little literature has investigated potential between-cities discrepancies in meteorological effects on HFMD occurrences. In this study, both the exposure-response curves and the heterogeneity analysis indicated significant geographical differences in meteorological effects, which were significantly associated with the latitude and longitude with an explained heterogeneity of 31.38 %. We found that HFMD occurrences were generally more sensitive to temperature effects at lower latitude, but more sensitive to relative humidity effects at higher latitude. The effect discrepancies are mainly because the cities at lower attitudes generally have tropical climate and are also located nearby the Pacific Ocean, which may provide higher humid climate so that people living in these areas are less sensitive to the effects of increased relative humidity. Meanwhile, sea water helps to cool down for people living around the Pacific Ocean at lower latitude and increase their sensitivity to temperature to some extent. Additionally, HFMD occurrences were more sensitive to temperature and relative humidity effects at higher longitude, while the effects variations at lower longitude like Xuwen were narrow. The effect of relative humidity on HFMD in Guangning seems to be relatively higher than the effects in other cities when the humidity was below 50 % (Fig. 2b). The common parameter specifications were used to make the estimates comparable among cities but may not be optimal for Guangning. The daily average cases were 4.2 in Guangning, much less than that (30.7) in other seven cities. The small number of daily cases may lead to biased effect estimates in Guangning.

There are several limitations in the present study. Firstly, although the 5-year data in eight cities in Guangdong were used as possible as we can, the number of daily HFMD cases for subgroups in each city was too small to support subgroup analyses for meteorological effects. Secondly, we considered many geographic, demographic, and socio-economic factors at the city level but the situations are relatively homogeneous in the same province and there may be other HFMD-related information, such as the level of health care utilization, which would help to explore the potential explanation for heterogeneity. Further analyses for a diverse range of cities in whole China with varying socio-economic conditions would provide an overall understanding of meteorological effects on HFMD occurrences in China, which may be very valuable for the prevention and control of HFMD.

## Conclusions

HFMD remains a crucial public health problem in Guangdong, China. HFMD cases clustered in male and children under 3 years old. Meteorological factors including temperature and relative humidity had delayed and non-linear effects on HFMD occurrences. The meteorological effects were closely linked to geographic longitude and latitude. These findings provide important evidence for further understanding of geographic variations of HFMD. The results can also help local authorities take corresponding interventions and measures to control HFMD development before reaching the peak risks. Further combination of HFMD data from other regions with more city-level information is warranted to construct the HFMD warning system.

## Additional files

Additional file 1: Spearman rank analysis between HFMD cases and meteorological factors in eight cities in Guangdong, 2009-2013. ** denotes significant correlations at the 0.01 level, and * represent significant correlations at the 0.05 level. (DOCX 20 kb) Additional file 2: The cumulative effects of sunshine and wind speed on HFMD over lag 0–14 days in Guangdong, 2009–2013. The bold red line represents the pooled effects, and the dashed lines represent the city-specific estimates. Reference values were the medians, that is, 4.78 h for sunshine and 2.70 m/s for wind speed. (DOC 52 kb) Additional file 3: Descriptive analysis for study-level variables in eight cities in Guangdong, 2009–2013. (DOCX 18 kb) Additional file 4: The stratified analyses of meteorological effects by seasons. The bold red line represents the pooled effects, and the dashed lines represent the city-specific estimates. Reference values were the medians, that is, 4.78 h for sunshine and 2.70 m/s for wind speed. (DOCX 1400 kb) Additional file 5: Daily aggregated data of HFMD cases and the demographic, socio-economic, and meteorological factors in each of eight cities in Guangdong, 2009–2013. The variables represent the date under study, city code, the number of daily HFMD cases, daily mean temperature, mean wind, mean air atmospheric pressure, mean humidity, rain, sunshine hour, and city-level information including longitude, latitude, GDP per capital, population, population density, the ratio of male to female population, day of the week, holiday, the ratio of children under 5 years old, living space per capita, the ratio of population flow. (CSV 1389 kb)

## Abbreviations

ARIMA: Autoregressive integrated moving average model
BLUP: Best linear unbiased prediction
China CDC: Chinese Centre for Disease Control and Prevention
CI: Confidence interval
*CV-A16*: *Coxsackievirus A16*
DLNM: Distributed lag non-linear model
*EV71*: *Enterovirus71*
GAM: Generalized additive model
HFMD: Hand, foot, and mouth disease
MGAM: Mixed generalized additive model
Q-AIC: Likelihood Akaike information criteria for quasi-Poison
REML: Restricted maximum likelihood
RR: Relative risk
SARIMA: Seasonal autoregressive integrated moving average

## References

[1] Zou XN, Zhang XZ, Wang B, Qiu YT (2012). Etiologic and epidemiologic analysis of hand, foot, and mouth disease in Guangzhou city: a review of 4,753 cases. Braz J Infect Dis.

[2] World Health Organization. Hand, foot, and mouth disease surveillance summary. Available at: http://www.wpro.who.int/emerging_diseases/HFMD/en/. Accessed 1st Aug 2015.

[3] Xing W, Liao Q, Viboud C, Zhang J, Sun J, Wu JT (2014). Hand, foot, and mouth disease in China, 2008–12: an epidemiological study. Lancet Infect Dis.

[4] Ang LW, Koh BK, Chan KP, Chua LT, James L, Goh KT (2009). Epidemiology and control of hand, foot and mouth disease in Singapore, 2001-2007. Ann Acad Med Singapore.

[5] Podin Y, Gias ELM, Ong F, Leong Y-W, Yee S-F, Yusof MA (2006). Sentinel surveillance for human enterovirus 71 in Sarawak, Malaysia: lessons from the first 7 years. BMC Public Health.

[6] Chang HL, Chio CP, Su HJ, Liao CM, Lin CY, Shau WY (2012). The association between enterovirus 71 infections and meteorological parameters in Taiwan. PLoS One.

[7] Lee C-CD, Tang J-H, Hwang J-S, Shigematsu M, Chan T-C. Effect of meteorological and geographical factors on the epidemics of hand, foot, and mouth diseasein island-type territory, East Asia. J Biomed Biotechnol. 2014;23. 10.1155/2015/805039 PMC4531172 26290875

[8] Wu H, Wang H, Wang Q, Xin Q, Lin H (2014). The effect of meteorological factors on adolescent hand, foot, and mouth disease and associated effect modifiers. Glob Health Action.

[9] Yu L, Zhou L, Tan L, Jiang H, Wang Y, Wei S (2014). Application of a new hybrid model with seasonal auto-regressive integrated moving average (ARIMA) and nonlinear auto-regressive neural network (NARNN) in forecasting incidence cases of HFMD in Shenzhen, China. PLoS One.

[10] Wei J, Hansen A, Liu Q (2015). The effect of meteorological variables on the transmission of hand, foot and mouth disease in four major cities of shanxi province, china: a time series data analysis (2009-2013). PLoS Negl Trop Dis.

[11] Chen C, Lin H, Li X, Lang L, Xiao X, Ding P (2014). Short-term effects of meteorological factors on children hand, foot and mouth disease in Guangzhou, China. Int J Biometeorol.

[12] Xie Y-h, Chongsuvivatwong V, Tang Z, McNeil EB, Tan Y (2014). Spatio-temporal clustering of hand, foot, and mouth disease at the county level in Guangxi, China. PLoS One.

[13] Ma W, Sun X, Song Y, Tao F, Feng W, He Y (2013). Applied mixed generalized additive model to assess the effect of temperature on the incidence of bacillary dysentery and its forecast. PLoS One.

[14] Gasparrini A, Armstrong B, Kenward MG (2010). Distributed lag non-linear models. Stat Med.

[15] Guo C, Yang L, Ou CQ, Li L, Zhuang Y, Yang J (2015). Malaria incidence from 2005-2013 and its associations with meteorological factors in Guangdong, China. Malar J.

[16] Hii YL, Rocklöv J, Ng N (2011). Short term effects of weather on hand, foot and mouth disease. PLoS One.

[17] Jackson D, Riley R, White IR (2011). Multivariate meta-analysis: potential and promise. Stat Med.

[18] Gasparrini A, Armstrong B, Kenward MG (2012). Multivariate meta-analysis for non-linear and other multi-parameter associations. Stat Med.

[19] Deng T, Huang Y, Yu S, Gu J, Huang C, Xiao G (2013). Spatial-temporal clusters and risk factors of hand, foot, and mouth disease at the district level in Guangdong Province, China. PLoS One.

[20] World Health Organization. Western Pacific Regional Office of the World Health. Available at: http://www.wpro.who.int/emerging_diseases/HFMD/en/index.html. Accessed 1st Aug 2015.

[21] Kim SJ, Kim JH, Kang JH, Kim DS, Kim KH, Kim KH (2013). Risk factors for neurologic complications of hand, foot and mouth disease in the Republic of Korea, 2009. J Korean Med Sci.

[22] Onozuka D, Hashizume M (2011). The influence of temperature and humidity on the incidence of hand, foot, and mouth disease in Japan. Sci Total Environ.

[23] Chen K-T, Chang H-L, Wang S-T, Cheng Y-T, Yang J-Y (2007). Epidemiologic features of hand-foot-mouth disease and herpangina caused by enterovirus 71 in Taiwan, 1998–2005. Pediatrics.

[24] Chan KP, Goh KT, Chong CY, Teo ES, Lau G, Ling AE (2003). Epidemic hand, foot and mouth disease caused by human enterovirus 71, Singapore. Emerg Infect Dis.

[25] Ishimaru Y, Nakano S, Yamaoka K, Takami S (1980). Outbreaks of hand, foot, and mouth disease by enterovirus 71. High incidence of complication disorders of central nervous system. Arch Dis Child.

[26] Zhu Z, Zhu S, Guo X, Wang J, Wang D, Yan D (2010). Retrospective seroepidemiology indicated that human enterovirus 71 and coxsackievirus A16 circulated wildly in central and southern China before large-scale outbreaks from 2008. Virol J.

[27] Chang LY, King CC, Hsu KH, Ning HC, Tsao KC, Li CC (2002). Risk factors of enterovirus 71 infection and associated hand, foot, and mouth disease/herpangina in children during an epidemic in Taiwan. Pediatrics.

[28] Li T, Yang Z, Di B, Wang M (2014). Hand-foot-and-mouth disease and weather factors in Guangzhou, southern China. Epidemiol Infect.

[29] Huang Y, Deng T, Yu S, Gu J, Huang C, Xiao G (2013). Effect of meteorological variables on the incidence of hand, foot, and mouth disease in children: a time-series analysis in Guangzhou, China. BMC Infect Dis.

[30] Lu P, Zhou B (2015). Hand-foot-mouth disease 17. Radiology of Infectious Diseases.

[31] Arita M, Shimizu H, Nagata N (2005). Temperature-sensitive mutants of enterovirus 71 show attenuation in cynomolgus monkeys. J Gen Virol.

[32] Stanton GJ, Langford M, Baron S (1977). Effect of interferon, elevated temperature, and cell type on replication of acute hemorrhagic conjunctivitis viruses. Infect Immun.

[33] Yu C-K, Chen C-C, Chen C-L (2000). Neutralizing antibody provided protection against enterovirus type 71 lethal challenge in neonatal mice. J Biomed Sci.

[34] Sheikh S. Cloning and nucleotide sequence analysis of the P3 region of enterovirus 70. University of Ottawa (Canada); 1990

[35] Lee C-CD, Tang J-H, Hwang J-S, Shigematsu M, Chan T-C (2015). Effect of meteorological and geographical factors on the epidemics of hand, foot, and mouth disease in island-type territory, East Asia. Biomed Res Int.

[36] Hu M, Li Z, Wang J, Jia L, Liao Y, Lai S (2012). Determinants of the incidence of hand, foot and mouth disease in China using geographically weighted regression models. PLoS One.

