# Supplementary material for: Short-term effects of meteorological factors on pediatric hand, foot, and mouth disease in Guangdong, China: a multi-city time-series analysis
Source: BMC Infect Dis. 2016 Sep 29;16:524. doi: 10.1186/s12879-016-1846-y (PMC5041518; doi:10.1186/s12879-016-1846-y)
Supplement: Additional file 1: — Spearman rank analysis between HFMD cases and meteorological factors in eight cities in Guangdong, 2009-2013. ** denotes significant correlations at the 0.01 level, and * represent significant correlations at the 0.05 level. (DOCX 20 kb) [file 12879_2016_1846_MOESM1_ESM.docx]

| Cities | Factors | Frequency | Temperature | Humidity | Pressure | Precipitation |
| --- | --- | --- | --- | --- | --- | --- |
| Guangning | Frequency | 1.000 | 0.490^**^ | 0.198^**^ | -0.486^**^ | 0.165^**^ |
|  | Temperature | 0.490^**^ | 1.000 | 0.076^**^ | -0.884^**^ | 0.054^*^ |
|  | Humidity | 0.198^**^ | 0.076^**^ | 1.000 | -0.285^**^ | 0.681^**^ |
|  | Pressure | -0.486^**^ | -0.884^**^ | -0.285^**^ | 1.000 | -0.262^**^ |
|  | Precipitation | 0.165^**^ | 0.054^*^ | 0.681^**^ | -0.262^**^ | 1.000 |
| Guangzhou | Frequency | 1.000 | 0.554^**^ | 0.315^**^ | -0.643^**^ | 0.199^**^ |
|  | Temperature | 0.554^**^ | 1.000 | 0.091^**^ | -0.821^**^ | 0.093^**^ |
|  | Humidity | 0.315^**^ | 0.091^**^ | 1.000 | -0.402^**^ | 0.647^**^ |
|  | Pressure | -0.643^**^ | -0.821^**^ | -0.402^**^ | 1.000 | -0.294^**^ |
|  | Precipitation | 0.199^**^ | 0.093^**^ | 0.647^**^ | -0.294^**^ | 1.000 |
| Heyuan | Frequency | 1.000 | 0.429^**^ | 0.195^**^ | -0.416^**^ | 0.175^**^ |
|  | Temperature | 0.429^**^ | 1.000 | 0.127^**^ | -0.865^**^ | 0.184^**^ |
|  | Humidity | 0.195^**^ | 0.127^**^ | 1.000 | -0.355^**^ | 0.784^**^ |
|  | Pressure | -0.416^**^ | -0.865^**^ | -0.355^**^ | 1.000 | -0.382^**^ |
|  | Precipitation | 0.175^**^ | 0.184^**^ | 0.784^**^ | -0.382^**^ | 1.000 |
| Luoding | Frequency | 1.000 | 0.366^**^ | 0.208^**^ | -0.343^**^ | 0.090^**^ |
|  | Temperature | 0.366^**^ | 1.000 | -0.049^*^ | -0.884^**^ | 0.059^*^ |
|  | Humidity | 0.208^**^ | -0.049^*^ | 1.000 | -0.149^**^ | 0.630^**^ |
|  | Pressure | -0.343^**^ | -0.884^**^ | -0.149^**^ | 1.000 | -0.242^**^ |
|  | Precipitation | 0.090^**^ | 0.059^*^ | 0.630^**^ | -0.242^**^ | 1.000 |
| Shantou | Frequency | 1.000 | 0.596^**^ | 0.167^**^ | -0.538^**^ | 0.093^**^ |
|  | Temperature | 0.596^**^ | 1.000 | 0.164^**^ | -0.861^**^ | 0.018 |
|  | Humidity | 0.167^**^ | 0.164^**^ | 1.000 | -0.396^**^ | 0.606^**^ |
|  | Pressure | -0.538^**^ | -0.861^**^ | -0.396^**^ | 1.000 | -0.265^**^ |
|  | Precipitation | 0.093^**^ | 0.018 | 0.606^**^ | -0.265^**^ | 1.000 |
| Shaoguan | Frequency | 1.000 | 0.405^**^ | 0.088^**^ | -0.432^**^ | 0.143^**^ |
|  | Temperature | 0.405^**^ | 1.000 | -0.082^**^ | -0.885^**^ | -0.043 |
|  | Humidity | 0.088^**^ | -0.082^**^ | 1.000 | -0.153^**^ | 0.724^**^ |
|  | Pressure | -0.432^**^ | -0.885^**^ | -0.153^**^ | 1.000 | -0.185^**^ |
|  | Precipitation | 0.143^**^ | -0.043 | 0.724^**^ | -0.185^**^ | 1.000 |
| Xuwen | Frequency | 1.000 | 0.172^**^ | -0.041 | -0.112^**^ | -0.028 |
|  | Temperature | 0.172^**^ | 1.000 | -0.306^**^ | -0.867^**^ | 0.014 |
|  | Humidity | -0.041 | -0.306^**^ | 1.000 | 0.086^**^ | 0.459^**^ |
|  | Pressure | -0.112^**^ | -0.867^**^ | 0.086^**^ | 1.000 | -0.204^**^ |
|  | Precipitation | -0.028 | 0.014 | 0.459^**^ | -0.204^**^ | 1.000 |
| Yangjiang | Frequency | 1.000 | 0.639^**^ | 0.184^**^ | -0.562^**^ | 0.187^**^ |
|  | Temperature | 0.639^**^ | 1.000 | 0.185^**^ | -0.849^**^ | 0.115^**^ |
|  | Humidity | 0.184^**^ | 0.185^**^ | 1.000 | -0.427^**^ | 0.633^**^ |
|  | Pressure | -0.562^**^ | -0.849^**^ | -0.427^**^ | 1.000 | -0.374^**^ |
|  | Precipitation | 0.187^**^ | 0.115^**^ | 0.633^**^ | -0.374^**^ | 1.000 |

### Additional file 1. Spearman rank analysis between HFMD cases and meteorological factors in eight areas in Guangdong, 2009-2013.
