# Supplementary material for: Short-term effects of meteorological factors on pediatric hand, foot, and mouth disease in Guangdong, China: a multi-city time-series analysis
Source: BMC Infect Dis. 2016 Sep 29;16:524. doi: 10.1186/s12879-016-1846-y (PMC5041518; doi:10.1186/s12879-016-1846-y)
Supplement: Additional file 2: — The cumulative effects of sunshine and wind speed on HFMD over lag 0–14 days in Guangdong, 2009–2013. The bold red line represents the pooled effects, and the dashed lines represent the city-specific estimates. Reference values were the medians, that is, 4.78 h for sunshine and 2.70 m/s for wind speed. (DOC 52 kb) [file 12879_2016_1846_MOESM2_ESM.doc]

Additional file 2


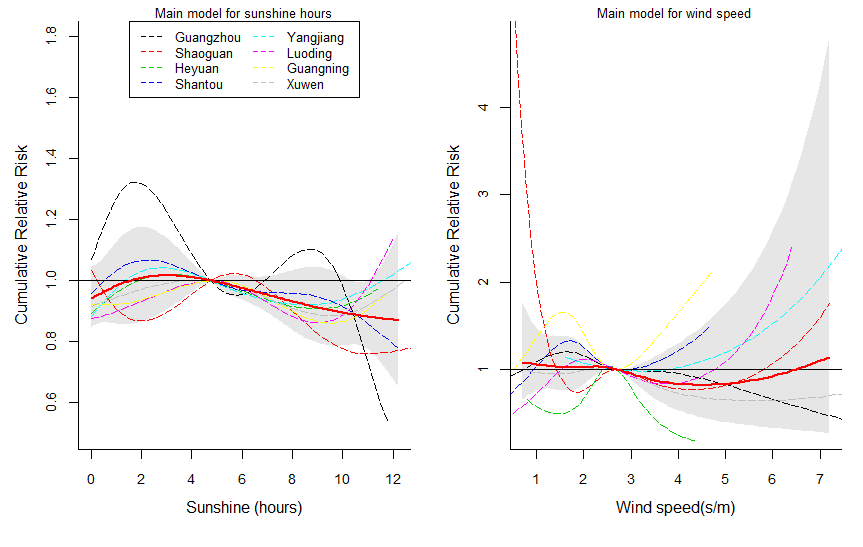


**The cumulative effects of sunshine and wind speed on HFMD over lag 0-14 days in Guangdong, 2009-2013**

The bold red line represents the pooled effects, and the dashed lines represent the city-specific estimates. Reference values were the medians, that is, 4.78 hours for sunshine and 2.70 m/s for wind speed.
