# Supplementary material for: Short-term effects of meteorological factors on pediatric hand, foot, and mouth disease in Guangdong, China: a multi-city time-series analysis
Source: BMC Infect Dis. 2016 Sep 29;16:524. doi: 10.1186/s12879-016-1846-y (PMC5041518; doi:10.1186/s12879-016-1846-y)
Supplement: Additional file 3: — Descriptive analysis for study-level variables in eight cities in Guangdong, 2009–2013. (DOCX 18 kb) [file 12879_2016_1846_MOESM3_ESM.docx]

### Additional file 3. Descriptive analysis for study-level variables in eight areas in Guangdong, 2009-2013.

|  |  | Guangning | Guangzhou | Heyuan | Luoding | Shantou | Shaoguan | Xuwen | Yangjiang |
| --- | --- | --- | --- | --- | --- | --- | --- | --- | --- |
| Temperature | Minimum | 5.10 | 5.10 | 3.35 | 5.70 | 7.00 | 2.00 | 8.40 | 6.20 |
|  | 5th percentile | 9.10 | 10.10 | 8.33 | 9.80 | 12.30 | 6.50 | 14.00 | 11.54 |
|  | Median | 23.30 | 23.50 | 22.73 | 23.90 | 23.30 | 21.60 | 25.40 | 23.80 |
|  | 95th percentile | 30.00 | 30.27 | 29.25 | 29.70 | 30.00 | 29.60 | 30.00 | 29.00 |
|  | Maximum | 31.90 | 32.30 | 31.08 | 32.00 | 31.80 | 31.50 | 32.20 | 31.10 |
|  | Mean | 21.65 | 22.05 | 21.01 | 22.20 | 22.41 | 19.95 | 23.95 | 22.42 |
| Humidity | Minimum | 41.00 | 27.00 | 29.00 | 44.00 | 30.00 | 32.00 | 52.00 | 28.00 |
|  | 5th percentile | 61.00 | 52.00 | 54.18 | 64.00 | 53.00 | 59.00 | 67.00 | 51.35 |
|  | Median | 77.00 | 78.00 | 74.63 | 79.00 | 77.00 | 75.00 | 82.00 | 82.00 |
|  | 95th percentile | 93.00 | 93.65 | 90.50 | 93.00 | 92.00 | 95.00 | 94.00 | 96.00 |
|  | Maximum | 100.00 | 100.00 | 97.50 | 100.00 | 98.00 | 100.00 | 100.00 | 100.00 |
|  | Mean | 77.30 | 75.87 | 74.22 | 78.68 | 75.18 | 75.83 | 81.82 | 79.14 |
| Atmopheric pressure | Minimum | 987.90 | 986.50 | 976.58 | 984.30 | 988.00 | 980.50 | 980.00 | 977.00 |
|  | 5th percentile | 995.60 | 994.80 | 986.60 | 995.04 | 1002.60 | 988.50 | 995.24 | 991.90 |
|  | Median | 1005.70 | 1005.70 | 995.96 | 1004.80 | 1012.80 | 999.00 | 1003.90 | 1001.40 |
|  | 95th percentile | 1017.70 | 1016.87 | 1007.65 | 1016.90 | 1023.00 | 1011.50 | 1014.80 | 1011.77 |
|  | Maximum | 1025.00 | 1026.60 | 1015.35 | 1023.90 | 1030.20 | 1019.00 | 1022.00 | 1018.60 |
|  | Mean | 1006.00 | 1005.98 | 996.53 | 1005.28 | 1012.81 | 999.35 | 1004.35 | 1001.50 |
| Precipitation | Minimum | 0.00 | 0.00 | 0.00 | 0.00 | 0.00 | 0.00 | 0.00 | 0.00 |
|  | 5th percentile | 0.00 | 0.00 | 0.00 | 0.00 | 0.00 | 0.00 | 0.00 | 0.00 |
|  | Median | 0.05 | 0.00 | 0.09 | 0.00 | 0.00 | 0.05 | 0.05 | 0.05 |
|  | 95th percentile | 24.45 | 30.70 | 25.18 | 21.97 | 21.57 | 28.90 | 23.60 | 38.70 |
|  | Maximum | 135.30 | 214.70 | 111.65 | 331.30 | 129.30 | 189.20 | 359.70 | 361.00 |
|  | Mean | 4.25 | 5.13 | 4.54 | 3.97 | 3.53 | 4.68 | 4.57 | 7.00 |
| Frequency | Minimum | 0.00 | 0.00 | 0.00 | 0.00 | 0.00 | 0.00 | 0.00 | 0.00 |
|  | 5th percentile | 0.00 | 6.00 | 1.00 | 1.00 | 2.00 | 0.00 | 0.00 | 0.00 |
|  | Median | 2.00 | 85.00 | 9.00 | 11.00 | 16.00 | 8.00 | 1.00 | 10.00 |
|  | 95th percentile | 17.00 | 431.90 | 43.00 | 43.65 | 59.00 | 48.00 | 6.00 | 52.00 |
|  | Maximum | 40.00 | 688.00 | 84.00 | 148.00 | 135.00 | 106.00 | 13.00 | 108.00 |
|  | Mean | 4.22 | 132.73 | 14.02 | 15.63 | 21.66 | 13.72 | 1.56 | 15.74 |
