# Supplementary material for: Short-term effects of meteorological factors on pediatric hand, foot, and mouth disease in Guangdong, China: a multi-city time-series analysis
Source: BMC Infect Dis. 2016 Sep 29;16:524. doi: 10.1186/s12879-016-1846-y (PMC5041518; doi:10.1186/s12879-016-1846-y)
Supplement: Additional file 4: — The stratified analyses of meteorological effects by seasons. The bold red line represents the pooled effects, and the dashed lines represent the city-specific estimates. Reference values were the medians, that is, 4.78 h for sunshine and 2.70 m/s for wind speed. (DOCX 1400 kb) [file 12879_2016_1846_MOESM4_ESM.docx]

Additional file 4. The stratified analyses of meteorological effects by seasons

**
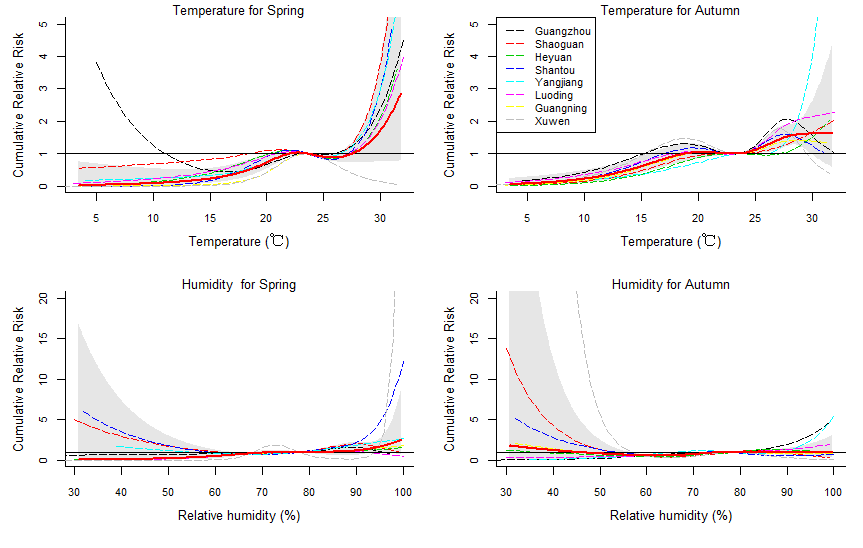
**

Figure S1. The stratified analyses of meteorological effects by seasons

Table S1. The cumulative pooled effects of meteorological factors on HFMD over 0-14 days

|  | Spring | Autumn |
| --- | --- | --- |
| Temperature (˚C, reference at 23.5 ˚C)^#^ | | |
| 9.8 (P5) | 0.08(0.01, 0.55)* | 0.21(0.10, 0.48)* |
| 17.4 (P25) | 0.39(0.21, 0.71)* | 0.88(0.64, 1.21) |
| 27.3 (P75) | 0.97(0.74, 1.28) | 1.47(1.16, 1.85)* |
| 29.7 (P95) | 1.60(0.75, 3.39) | 1.61(1.07, 2.42)* |
| Relative humidity (%, reference at 78%)^#^ | | |
| 57.0 (P5) | 0.37(0.08, 1.64) | 0.60(0.41, 0.90)* |
| 70.3 (P25) | 0.91(0.70, 1.17) | 0.73(0.56, 0.96)* |
| 85.0 (P75) | 1.01(0.59, 1.73) | 0.96(0.74, 1.25) |
| 94.0 (P95) | 1.51(0.98, 2.33) | 0.93(0.46, 1.86) |

^#^The reference value is the median for each meteorological measures.

*Confidence intervals that do not overlap the null value of RR=1.
